# A generalizable NLP framework for fast development of pattern-based biomedical relation extraction systems

**DOI:** 10.1186/1471-2105-15-285

**Published:** 2014-08-23

**Authors:** Yifan Peng, Manabu Torii, Cathy H Wu, K Vijay-Shanker

**Affiliations:** Department of Computer and Information Sciences, University of Delaware, 18 Amstel Ave, Newark, DE 19716 USA; Center for Bioinformatics and Computational Biology, University of Delaware, 15 Innovation Way, Newark, DE 19711 USA

## Abstract

**Background:**

Text mining is increasingly used in the biomedical domain because of its ability to automatically gather information from large amount of scientific articles. One important task in biomedical text mining is relation extraction, which aims to identify designated relations among biological entities reported in literature. A relation extraction system achieving high performance is expensive to develop because of the substantial time and effort required for its design and implementation. Here, we report a novel framework to facilitate the development of a pattern-based biomedical relation extraction system. It has several unique design features: (1) leveraging syntactic variations possible in a language and automatically generating extraction patterns in a systematic manner, (2) applying sentence simplification to improve the coverage of extraction patterns, and (3) identifying referential relations between a syntactic argument of a predicate and the actual target expected in the relation extraction task.

**Results:**

A relation extraction system derived using the proposed framework achieved overall F-scores of 72.66% for the Simple events and 55.57% for the Binding events on the BioNLP-ST 2011 GE test set, comparing favorably with the top performing systems that participated in the BioNLP-ST 2011 GE task. We obtained similar results on the BioNLP-ST 2013 GE test set (80.07% and 60.58%, respectively). We conducted additional experiments on the training and development sets to provide a more detailed analysis of the system and its individual modules. This analysis indicates that without increasing the number of patterns, simplification and referential relation linking play a key role in the effective extraction of biomedical relations.

**Conclusions:**

In this paper, we present a novel framework for fast development of relation extraction systems. The framework requires only a list of triggers as input, and does not need information from an annotated corpus. Thus, we reduce the involvement of domain experts, who would otherwise have to provide manual annotations and help with the design of hand crafted patterns. We demonstrate how our framework is used to develop a system which achieves state-of-the-art performance on a public benchmark corpus.

## Background

Due to the continued growth of biomedical publications, it has become very difficult for scientists to keep up with the new findings reported in the literature. As a consequence, we have observed an increase in the effort spent on automatically extracting information from research literature and developing biomedical text mining tools.

This paper aims to address the relation extraction task, which identifies selected types of relationships among entities (e.g., proteins) reported in text.

Approaches to the relation extraction task can be categorized into two major classes: (1) machine learning-based approaches and (2) pattern-based approaches. Machine learning-based approaches are data-driven and can derive models from a set of annotated data [1–7]. The use of machine learning methods can be quite effective, but the performance of resulting systems depends on the quality and the amount of annotated data. For example, large annotated corpora become available for the protein-protein interaction relation task, reflecting a general community-wide interest [8]. But this situation does not always hold for relations of different scientific interest, because preparing annotated corpora is generally time consuming and expensive and it also requires domain expertise and significant effort to ensure accuracy and consistency. In contrast, pattern-based approaches do not require annotated data to train a system. However, they do require domain experts to be closely involved in the design and implementation of the system to capture the patterns used for extracting the necessary information. Some systems rely on extraction patterns defined at the surface textual level or based on outputs from a shallow parser [9–12]. Others use deep parsers with hand-crafted patterns [13–17]. As found in OpenDMAP [18], a semantic grammar may be utilized with text literals, syntactic constituents, semantic types of entities, and hyponomy. In all cases, rigid extraction patterns are manually encoded in the systems. Owing to rigid patterns, pattern-based approaches usually achieve a high precision but are often cited for low recall. While it is feasible to manually identify and implement high quality patterns to achieve a good precision, it is often impractical to exhaustively encode all the patterns necessary for a high recall in this manner.

Our work enables the fast development of pattern-based systems, while mitigating some of these concerns. We aim to reduce the involvement of domain experts and their manual annotation, and to attain high precision and recall.

Our approach starts by identifying a list of trigger words for the target relation (e.g., “associate” for the binding relation) and their corresponding Trigger specifications (e.g., the number and type of arguments expected for each trigger). Given this information, we make use of linguistic principles to derive variations of lexico-syntactic patterns in a systematic manner. These patterns are matched with the input text in order to extract target relations.

To improve the applicability of the generated patterns, we incorporate two additional design features. The first is the use of text simplification. This allows us to design a small set of lexico-syntactic patterns to match simple sentence constructs, rather than try to account for all complex syntactic constructs by generating an exhaustively large amount of patterns. Second, the framework exploits referential relations. With this method, two phrases referring to the same entity (e.g., coreference relation) or in a particular relation (e.g., meronymy relation, also known as part-of relation) are detected in text, and links are established between them. These links can be used when seeking the most appropriate phrase referring to the target entity and, hence, allow for extraction of target entities beyond lexico-syntactic patterns.

The proposed approach is based on the property of the language, rather than task-specific knowledge. Therefore, it is generalizable for different trigger words and potentially applicable to many different types of information targeted in biomedical relation extraction tasks.

We acknowledge several studies underlying our proposed framework. The automated pattern generation employed in this study shares the fundamental assumptions of certain linguistic theories, such as Lexicalized Tree Adjoining Grammar (LTAG) [19], Head-Driven Phrase Structure Grammar [20], and Lexical Functional Grammar [21]. In particular, we believe that the concept underlying our method is similar to that of LTAG. The paradigm of inferring patterns exploited in our method shares the ideas with [22–30], but we focus on a specific set of patterns pertaining to the expression of biomedical relations.

Simplifying a sentence as a prerequisite for biomedical information extraction was studied in the past [9, 11, 31–34]. The use of meronymy and its opposite holonymy, among other relationships found in the biomedical ontology, was discussed in [35]. Some of these relations were later considered in biomedical information extraction systems in order to improve their performance [36–38]. These relations and paradigms are in conjunction with our own two additional referential relationships: coreference and hyponymy. We integrate them in our framework and examine their utility for biomedical relation extraction.

To evaluate the framework, we test it by producing an extraction system for six relations that were part of the BioNLP-ST 2011 and 2013 GE tasks. We show that by just taking the specification of trigger words (root word only), we produce a relation extraction system with results that compare favorably with state-of-the-art results on this corpus. We further show that we can achieve good precision and recall with the patterns generated from the trigger, and that simplification and referential relation linking can increase the recall without compromising the precision.

## Methods

### A. Architecture overview

**Figure 1:**
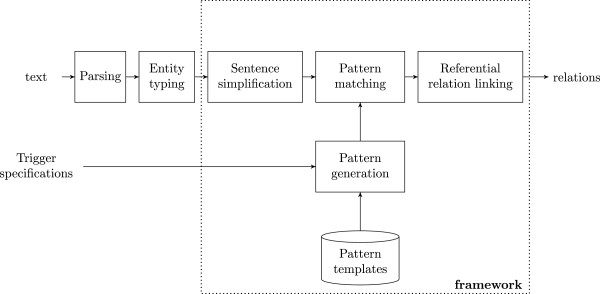
**Framework architecture.** The framework consists of four modules: Pattern generation, Pattern matching, Sentence simplification, and Referential relation linking. There are two external modules: Parsing and Entity typing. The framework requires Trigger specifications and pre-defined Pattern templates to generate patterns. Then it extracts *relations* from *text* using the generated patterns.

The architecture of our framework has several components (Figure 1), as summarized below and detailed in sections B-F. The framework consists of four system modules (Pattern generation, Pattern matching, Sentence simplification, and Referential relation linking) and two external modules (Parsing and Entity typing).

It also requires *Trigger specifications* and associated *Pattern templates* to locate the relations of interest. An example trigger specification is shown below:


In the above example, Line 1 shows the trigger word, “phosphorylate” in this case. Line 2 indicates that it is the trigger for the relation “phosphorylation”. Line 3 specifies that the trigger syntactically chooses two noun phrases, designated as NP _0_ and NP _1_. Lines 4–5 add selectional restrictions, by requiring NP _0_ to be a gene or gene product (GGP) and NP _1_ to be either a GGP or a protein part. Lines 6–7 show that if NP _0_ and NP _1_ can be extracted, and if both NP _0_ and NP _1_ meet the above constraints, then the framework will assign their semantic roles of agent and theme, respectively.

Now consider the following example sentence:

(2) The c-Jun amino-terminal kinase **phosphorylates***NFAT4*.

From (2), we will extract “the c-Jun amino-terminal kinase” as the agent and “NFAT4” as the theme of the phosphorylation relation. This extraction is the result of matching the text fragment with a pattern that is partly derived from the trigger specification. This pattern should not only capture the general syntactic form of a clause involving a transitive verb in an active voice, but also capture the selection restrictions imposed by the word “phosphorylates” and the arguments. Thus, this pattern contains information described in two places: (1) lexical trigger that specifies the arguments, the selection restrictions on the argument, and the role they play, and (2) the syntactic constraints corresponding to different constructs (in this example, the active clause). We call the former “trigger specification”, and the latter “pattern templates”. Actual lexico-syntactic patterns are obtained by merging the trigger specifications and pattern templates. As we shall see later (section B), the combination of these two is mediated by the frame that is mentioned in the trigger specification.

We now briefly discuss four modules of the system framework: Pattern generation, Pattern matching, Sentence simplification, and Referential relation linking (Figure 1).

The Pattern generation (section C) module uses trigger specifications and predefined pattern templates to derive lexico-syntactic patterns for each trigger word. The Pattern matching (section D) module then matches fragments of text with lexico-syntactic patterns, and extracts the textual expressions in the trigger and argument positions. In order to more effectively match with the patterns, the Sentence simplification (section E) module is used to preprocess the input text. It aims to ensure that the lexico-syntactic patterns generated in the previous step are able to be matched even in complex sentences. Finally, the Referential relation linking (section F) module links arguments identified by the pattern matching module with the target entities they refer to, where applicable. This step enables the system to find relations between more appropriate entities than the ones referred by textual expressions in the argument position.

In addition to the above four system modules, there are two external modules. One is the *Parsing* module, which is used by the pattern matching step. The other is the *Entity typing* module, which assigns semantic types or categories to noun phrases. Both are found to be useful to enhance the precision of the relation extraction task [12, 18, 39].

### B. Trigger specification

Trigger specifications are used to locate triggers and arguments in text for target relations. To make it easier to specify triggers, we ask users to provide the trigger’s root, which is the primary lexical unit of a word. From the root morpheme, we can derive other forms of triggers using our previous work [40]. For example, from “phosphorylate” we derive “phosphorylates”, “phosphorylated”, “phosphorylation”, etc. In general, we generate different possible forms of triggers and confirm whether they are used in the literature. In a few cases, we ask the users for this confirmation. This generation is based on well-known English inflection rules, and this can be used to match to the appropriate trigger template.

Next, we show two example trigger specifications for the same root morpheme, “express”, but with different semantic types for the argument, gene and RNA.


Although these two specifications share the same trigger word, they represent different types of relations: gene expression and transcription. The gene expression relation requires its theme (NP _1_) to be a gene, whereas the transcription relation requires its theme (NP _0_) to be an RNA. These examples show that argument types in the trigger specification are essential to the framework to achieve a high precision, because they emphasize the selection restrictions on arguments.

### C. Pattern generation

Provided with a trigger specification, we use the “frame” to associate a trigger with a set of pattern templates to derive lexico-syntactic patterns. In the following subsections, we will define frames and pattern templates, and then discuss how they can be combined to generate lexico-syntactic patterns.

#### C.1 Frames

A frame is a set of pattern templates sharing the same syntactic nature of the constituents that are likely to be associated with the trigger. It specifies the arguments of the trigger. We found that the most frequent frame in biomedical documents is:

(5) *Frame:NP*_0_*/NP*_1_

We distinguish NP _0_ and NP _1_, because semantically they play different roles and have different types in the trigger specification, and syntactically they represent different grammatical constituents. The above frame may be realized by the standard active form “NP _0_ V NP _1_”, where V is a verb, and NP _0_ and NP _1_ appear at the left and right of the verb, respectively.

Relations can be semantically “directional” or “non-directional”. For example, phosphorylation is a directional relation, but binding is non-directional. This is because “A binds B” and “B binds A” may be used to specify the same relation between “A” and “B”, but “A phosphorylates B” and “B phosphorylates A” represent two different relations. If a relation is directional (or non-directional), we would expect that all triggers for that relation have the property as well. In our framework, we use an additional binary constraint “ 〈*direction*〉= directional/non-directional” in the trigger specification to distinguish non-directional relations from the other, because currently it is the only place where users interact with the framework. To generate appropriate patterns, this directionality constraint in the trigger specification will cause an appropriate defined frame to be chosen: the non-directional frame differs from the directional one by allowing for the swapping of the agent and the theme.

#### C.2 Pattern templates

A pattern template is specified by a sequence of words or phrases *β*_1_,…,*β*_*n*_, followed by a set of constraints. Each constraint assigns a value for one of the *β*_*i*_ features.

To reduce the number of pattern templates, we limit pattern templates to capture one argument at a time. So the pattern templates will capture pairs <trigger, NP_i_ >. After templates are instantiated and arguments are extracted, we combine pairs if they have the same trigger. Thus we can extract relations with multiple arguments. We believe that considering one argument at a time is more flexible and manageable, because such pairs can be applied independently, while constraints on combinations can cover many different relations.

We further categorize pattern templates into two groups: one with explicit argument, and one with null argument. We will discuss pattern templates for argument realization in the next section, and then introduce methods to generate lexico-syntactic patterns. Lastly, we will discuss pattern templates with null argument.

#### C.3 Pattern templates for argument realization

Argument realization, which is at the heart of the area of linguistics, is the study of the possible syntactic expressions of the arguments of a verb [41]. In this study, we extend argument realization to nominal and adjectival triggers derived from verbs as well.

##### Verbal triggers

Below are examples of pattern templates for verbal predicate V_tr_ in active voice:


An example template for a verbal predicate V_tr_ in passive voice is:


We use NP_1_ in pattern templates (7) and (8) in contrast to NP_0_ in template (6), because their roles are different. For example, in trigger specification of (1), NP_0_ is always the agent and NP_1_ is always the theme. Furthermore, in combination with the constraints expressed within the trigger specification, the use of template (6) will succeed only if NP_0_ is GGP, whereas the use of template (8) will succeed even if NP_1_ is a protein part.

##### Nominal triggers

In addition to the standard pattern templates that are based on verbal forms of the trigger, we also consider cases where the trigger verb is nominalized (N_tr_). For example, “transcribe” can be nominalized into “transcription” or “transcript”. Nominalization of verbs can be divided into two classes. The first class is where resulting nouns denote actions, states, and processes. Their suffixes are typically “-ion”, “-age”, and “-ance” (e.g., “transcribe” →“transcription”, “cleave” →“cleavage”, and “appear” →“appearance”). The second class is where resulting nouns refer to entities (e.g., “transcribe” →“transcript” and “produce” →“product”). Because our primary interest is processes pertaining to genes or proteins, we currently focus on the first class. Typical pattern templates for nominal triggers are:


Besides the theme, the agent can be incorporated via a “by” phrase. A pattern template for such instances is:


As for non-directional relations we discussed earlier, we have additional templates, which are exemplified by the following:


##### Adjectival triggers

English has a general morphological process of adjective conversion (Adj_tr_), which enables verbs to be used as adjectives. The pattern template for adjective triggers is


In this framework, adjectival derivations can be the present participle (14a), the past participle (14b), and the adjectivization (14c) of a verb.


#### C.4 Generation of patterns

The pattern generation module automatically creates lexico-syntactic patterns given a list of trigger specifications and frames.

To associate pattern templates with frames, verb type information is used. For example, one constraint in English is that only transitive verbs can be passivized. Therefore *Frame:NP*_0_*/NP*_1_ contains template (6), (7), and (8), but *Frame:NP*_0_ contains template (6) only. Given the trigger specification of (1) for “phosphorylate” having *Frame:NP*_0_*/NP*_1_, we will automatically generate lexico-syntactic patterns like “NP_0_ phosphorylates”, “NP_1_ is phosphorylated”, etc..

The automatic generation procedure is similar to the concept of LTAG. In LTAG, a tree family associates a verb lexeme with a given subcategorization. The subcategorization includes a set of grammatical structures that represent all the possible lexico-syntactic variations for that verb lexeme. So grammatical structures can be obtained by combining lexical rules and syntactic transformations. Compared with LTAG, the “frame” in our approach is essential but not exactly a subcategorization in LTAG. The trigger specifications are similar to tree families in LTAG, which associate a trigger lexeme with a given frame. In addition, we also consider verb nominalization and adjectivization.

#### C.5 Pattern templates with null argument

There are cases when the writing style does not follow the common trigger-argument association. When the argument is omitted, but implied, we call them “elliptical construction”. Following are some examples of (a) elliptical constructions, and (b) how they would be written if the trigger-argument association would be required.


Both (a) and (b) are grammatically correct and express the same underlying idea in (15) and (16), but we tend to write (a) rather than (b) as a shorthand. Such null argument structure is similar to the null complement anaphora (deep anaphora) in a modern syntactic theory [42] and the implicit argument in a semantic theory [43]. For the relation extraction task, we observe that the elided argument may be found as its antecedent and determined by another trigger that selects it. Our framework recovers them as part of the relation extraction process, by applying for the null argument pattern templates. It should be noted that such elliptical constructions can appear in various positions of a sentence, e.g., at the beginning (15a) or at the end (16a). These templates always rely on the whole sentence construct, therefore are too cumbersome to express. We designed some pattern templates to match sentences like (15a) and (16a). Whether there exists a more general and clearer way to express these types of pattern templates needs to be further explored.

### D. Pattern matching

Consider the text fragment where “JNK” and “NFAT4” have already been tagged as gene or gene product.

(17) JNK phosphorylates NFAT4

This fragment is captured by the generated lexico-syntactic patterns derived from *Frame:NP*_0_*/NP*_1_ and the trigger specification “phosphorylate” in (1). The next step is to extract the actual agent and theme. Specifically, pattern template (7) matches the “phosphorylates” word as a trigger, and “NFAT4” as NP_1_. The trigger specification (1) checks NP_1_’s type, which is GGP, and assigns its role for a theme. Therefore, we get <phosphorylates_trigger_, NFAT4_theme_ >. Similarly, by using pattern template (6) we can extract <phosphorylates_trigger_, JNK_agent_ >.

For pattern matching, we would like to mention two issues. First, as illustrated above, the pattern matching engine must be able to check the types of NPs are consistent with those mentioned in the trigger specifications. For this purpose, any method that assigns types to noun phrases or named entities, such as BioNex [39] or Genia tagger [44] can be employed. In our evaluation, we have used the BioNex tool.

Secondly, in order to match a broader range of phrases, we skip verbal auxiliaries and adjuncts for pattern matching. By auxiliary verbs, we mean verbs used to express tense, aspect, modality, etc. By adjuncts, we mean the optional phrases that do not affect the main meaning of a sentence. First, consider the following examples having auxiliary verbs:


The above examples belong to pattern templates (7) and (8), respectively. However, none of them can be directly matched because of the complex way in which the predicates are expressed. This construction of consecutive verb groups makes basic pattern matching extremely laborious, because of the many variations they can introduce. In this framework, we would like to avoid constructing complex pattern templates, thereby reducing the burden of system development. We notice that (1) syntactically, such consecutive verb groups form a dependent-auxiliary construction: dependent-auxiliary + main-verb, and (2) semantically, the “agent” and “theme” are always related to the last main-verb, rather than the auxiliary. Thus, we match the consecutive verb group as a whole, then choose the last verb as the head of the whole sequence.

Second, let us look at adjuncts in the following sentences:


The most frequent adjuncts that are likely to be skipped are the adverbial adjuncts, e.g. “abundantly” and “in the granulocyte fraction of human peripheral blood cells” in (19a). In addition, adjective-nominal adjuncts are also skipped, e.g “Abundant” and “in human peripheral granurocytes” in (19b).

### E. Sentence simplification

So far, we have discussed how arguments can be extracted by matching patterns. But even with a large number of patterns automatically generated in the proposed manner, the recall of the resulting system is still low because sentence constructions and writing styles vary considerably in actual text, and the number of variations to be considered is overwhelmingly high. For example, consider sentence (20):

(20) Active *Raf-1***phosphorylates** and activates the mitogen-activated protein (MAP) kinase/extracellular signal-regulated kinase kinase 1 (*MEK1*), which in turn phosphorylates and activates the MAP kinases/ extracellular signal regulated kinases, *ERK1* and *ERK2*. (PMID 8557975)

It is difficult to extract phosphorylation relation <phosphorylates_trigger_, ERK1_theme_ > and <phosphorylates_trigger_, ERK2_theme_ > from (20) by preconceiving complex patterns required and exhaustively encoding them along with all possible variations. On the other hand, if we can simplify the syntactic structure of (20) and obtain the following sentences, the automatically generated patterns can easily match the simple sentences:


This and many other instances that we observed in biomedical research articles motivated us to separate the various structures of a sentence first, and then match patterns to the simplified sentences.

Complex constructs, e.g., coordinations and relative clauses, pose a challenge for state-of-the-art full parsers. However, even if these constructs can be detected correctly using full parsers, new patterns are still needed to skip parts of a construct (e.g., skipping conjuncts in a coordination or skipping relative clauses). When using a dependency parser, more collapsed rules involving prepositions, conjuncts, as well as information about the referent of relative clauses are used to get direct dependencies between content words [45]. Both cases will increase the complexity of patterns and, thus, increase the pattern encoding effort.

Alternatively in this framework, we introduce sentence simplification as a preprocessing module. Given an input sentence, this module outputs a set of generated simplified sentences, thus conceals the syntactic complexity from the pattern matching step.

#### E.1 Complex constructs for simplification

In this section, we will describe syntactic constructs that the preprocessing module simplifies. For further details of our sentence simplifier, iSimp, we refer to [33].

Coordinations are syntactic structures that link two or more items (conjuncts) of syntactically equal status [46]. These conjuncts are linked by coordinating conjunctions (e.g., “and”, “or”, and “but”). Our primary concerns are coordinations of nouns (22a), noun phrases (22b), verbs (22c), and verb phrases (22d).


For a coordination, the original sentence can be split into multiple ones, each containing one conjunct.

Relative clauses are clauses that modify noun phrases. For example,


There are two types of relative clauses that frequently appear in biomedical text: full relative clauses and reduced relative clauses. Full relative clauses (23a) are introduced by relative pronouns, such as “which”, “who”, and “that”. Reduced relative clauses (23b) and (23c) start with a gerund or past participle and have no overt subject. A sentence containing a relative clause can be simplified into two sentences: the original sentence without the relative clause and the other that consists of referred noun phrase as a subject and the relative clause.

Appositions are constructs of two noun phrases next to each other, typically separated by comma and referring to the same entity. For example,


Appositions can be detected by searching for two noun phrases separated by a comma, when they are not part of a coordination. In addition, because one noun phrase (appositive) normally renames or describes the other, it usually begins with a determiner or a number (as shown above). Appositions can be simplified into two sentences: one with the referred noun phrase and the other with the appositive.

Parenthesized elements are any words enclosed within “()”, “[]”, and “{}”. They usually refer to or describe preceding noun phrases.


When simplifying parenthesized elements, an additional sentence is created only with the parenthesized elements without the preceding nouns phrase.

#### E.2 Dealing with attachment ambiguities

Attachment of phrases poses one of the well-known problems in syntactic ambiguity.


Examples in (26) refer to relative clause attachment ambiguities, where there is a complex NP of the type “NP_1_*prep* NP_2_” followed by a relative clause. In such cases, it is unclear whether to attach the relative clause to the first noun phrase (NP_1_) or the second one (NP_2_). Other kinds of attachment ambiguity include PP-attachment, e.g., “NP_1_ and NP_2_ PP” (27), and the attachment involving coordination, e.g., “Adj NP_1_ and NP_2_” (28). Solving the attachment problem is important in sentence simplification, but we believe it is not a purely syntactic problem [47]. Semantic information is also necessary to make a decision. Therefore, in this study, we produce alternative attachments as candidates while simplifying sentences, and leave the decision to the pattern matching module where type information is available.

### F. Referential relation linking

By using patterns and sentence simplification, the system can detect textual expressions in the argument position. Sometimes, the referred entity is mentioned somewhere else in the text. Consider example (29). The system can extract binding relation <dimerized_trigger_, the protein_theme_ > from (29), but the actual target entity is “c-Fox”. To link these phrases, we developed patterns to extract referential relations.

(29) The stability of *c-Fox* was decreased when *the protein***was dimerized** with phosphorylated c-Jun.

#### F.1 Referential relations

Referential relation patterns are designed to extract the relationship of one nominal phrase to another, when one provides the necessary information to interpret the other [48]. By utilizing referential relations, an extraction system is able to identify an actual target entity beyond the initially extracted arguments.

Co-referential relations (or co-references) occur when multiple expressions refer to the same referent. For instance, in the previous example, “the protein” and “c-Fox” both refer to the same object. In a co-referential relation, the anaphoric reference can be a pronoun or definite noun phrase, and its antecedent can be the actual name of protein or gene. In this study, co-referential relations are not extracted, except for the case of a relative pronoun, because we consider their detection as a separate and independent task from pattern-based extraction.

Part-whole relations are useful when an argument extracted for a trigger comprises a part of the target entity. For example:

(30) Both Eomes and Runx3 **bind** at the *Prf1***locus**.

For biomedical information extraction, this framework focuses on relations between protein parts and a protein, e.g., a residue in a protein. Such part-whole relations in example (30) can be captured by patterns like “NP_whole_*contains* NP_part_” or by the existence of keywords like “locus”, “promoter”, and “domain”.

Member-collection relations are useful in linking a generic reference to a group of entities that are specified in other places in text. For example:

(31) **expression** of *adhesion molecules***including***integrin alpha, L-selectin, ICAM-3*, and *H-CAM*

The above example illustrates that the generic reference “adhesion molecules” can be extracted as an argument of the trigger “expression”. Meanwhile, specific referred entities include “integrin alpha”, “L-selectin”, “ICAM-3” and “H-CAM”. We consider patterns like “NP, *such as* NP (, NP)*” to identify this type of relations.

Hyponymy relations are used when argument X is a hyponym of argument Y, if X is a subtype of Y, or when an instance of X refers to a concept Y. Thus, in (32a), “CD14” is said to be a hyponym of “membrane glycoprotein”, and in (32b), “p130 Crk-associated substrate (Cas)” is a hyponym of “protein”. When linked, the system extracts <expressed_trigger_, CD14_theme_ > and <phosphorylated_trigger_, Cas_theme_ >, respectively.


To achieve this goal, we identify the fragments having keywords such as “acts as” or “is identified as”, which are similar to the ones in [49] and [50]. Moreover, the apposition construct can also hold a hyponymy relation between the appositive and the referred noun phrase.

#### F.2 Linking entities through referential relations

**Figure 2:**
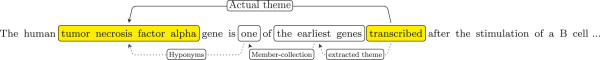
**An example of the referential relation linking.** The pattern will extract “the earliest genes” as the theme first. Then with the member-collection and hyponymy relations linking, the framework can identify “tumor necrosis factor alpha” as the actual theme of “transcribed”.

We will use the example in Figure 2 to illustrate integrating basic patterns and linking relations.

This example contains one transcription relation. Our goal is to extract its trigger and argument, namely <transcribed_trigger_, tumor necrosis factor alpha_theme_ > which are highlighted in the sentence. We assume “tumor necrosis factor alpha” is typed as a gene.

Given the trigger “transcribe” and using pattern template (8) as discussed earlier, we can extract <transcribed_trigger_, the earliest genes_theme_ >. But “the earliest genes” is not a named entity (This can be discovered by using a named entity recognition tool). In addition, we extract one member-collection relation <one_member_, the earliest genes_collection_ > and one hyponymy relation <tumor necrosis factor alpha_hyponym_, one_hypernym_ >. The first relation enables us to infer <transcribed_trigger_, one_theme_ >, since the collection of genes (“the earliest genes”) are “transcribed” and, then, one of its members can be “transcribed” as well. Then, the latter relation allows us to state “tumor necrosis factor alpha” is the “one” in this context and hence to conclude <transcribed_trigger_, tumor necrosis factor alpha_theme_ >.

The algorithm for the linking is as follows. First we collect all referential relations in the document. Then we use the patterns to get instances for a trigger. If the instance’s argument is not an informative reference, we recursively search for all of its references in the detected referential relations. If an appropriate reference of an entity is found, we link it to the trigger, by creating a new pair <trigger, referred entity >. This search procedure ends when we exhaust all possibilities. As a result, more than one pair may be created and all pairs are proposed.

### G. Evaluation design

Our framework is designed to extract a variety of relations. For the evaluation of our framework, we need test sets containing different types of relations. Furthermore, the data set should include trigger annotations needed to automatically generate patterns. We chose to use the corpora of BioNLP-ST (Shared Tasks) 2011 and 2013 GE tasks, which included several event extraction subtasks [51, 52].

#### G.1 BioNLP-ST GE task

The BioNLP-ST GE task series aim to extract various events from biomedical text. The first shared task workshop was held in 2009, and the most recent one in 2013. In this study, both 2011 and 2013 corpora are used for the evaluation. We will refer to them as “GE 2011” and “GE 2013” hereafter.

In GE 2011 task, evaluation results were reported on (W)hole, (A)bstract, and (F)ull paper collections, respectively. The abstract collection contains paper abstracts, the full text collection contains full papers, and the whole collection contains both abstracts and full text. Following the same setting, we also report our results on W, A, and F. GE 2011 corpus covers nine types of events: Gene_expression, Transcription, Localization, Protein_catabolism, Phosphorylation, Binding, Regulation, Positive_regulation, and Negative_regulation. Among these, we focused on events with simple entities as themes. Thus, Regulation and its subtypes were removed because their themes could be other events with other triggers. As a result, only the first 6 types of events were evaluated. The first five events were called “Simple Event” collectively. In the GE 2013 corpora, we consider the same events as well.

#### G.2 Trigger selection

Since our approach requires a list of triggers, we used the triggers annotated in the corpus. To effectively evaluate our framework, we further decided to focus on a selected group of triggers. Among triggers in GE corpus, we chose only the triggers that are based on verbs (e.g., phosphorylate) and their nominal and adjective forms (Table 1) as discussed before. We did not use the triggers that are pure nouns (e.g., level) or adjectives (e.g., positive). Additionally, we eliminated verb triggers like “find” and “form” because they are not specific to particular biomedical events.

**Table 1:** **Selected triggers**

Events	Verb	Derivation
Gene_expression	express	-ion, over-, co-, non-, re-
	produce	-ion, non-, co-
Transcription	express ^1^	*See above*
	initiate	-tion
	produce ^1^	*See above*
	transcribe	-tion, -tional, -tionally
Protein_catabolism	cleave	-age
	degrade	-tion, -tive
	proteolyse	-sis, -tic, -tically
Phosphorylation	phosphorylate	-ion, under-, hyper-
Localization	accumulate	-ation
	appear	-ance
	detect	
	export	
	express ^2^	*See above*
	import	
	localize	-ation, co-, re-
	locate	-ion, re-, trans-
	migrate	co-
	mobilize	-ation, im-
	release	
	secrete	-ion
	transport	
Binding	associate	-ion
	bind	DNA-
	engage	-ment
	interact	-ion
	ligate	-ion, co-
	link	cross-
	oligomerize	-ation
	recruit	-ment
	immunoprecipitate	co-

1. This predication is always used together with “mRNA”.

2. This predication is always used together with “surface”.

The *Derivation* column shows affix used to derive other forms of triggers. Singular, past tense, and gerund forms are not shown.

#### G.3 Evaluation measurement

The evaluation was carried out by comparing the predicted annotation with the gold standard. We used the approximate recursive matching decomposition mode as in the GE task [51], which requires extracting equality between the two events as follows:

1. the event types are the same;
2. the triggers are the same; and
3. the arguments are the same.

Same triggers and arguments means that “the given text span is equivalent to a gold span if it is entirely contained within an extension of the gold span by one word both to the left and to the right.” For example, if (*a*_1_,*b*_1_) is the given span and (*a*_2_,*b*_2_) is the gold span, they are the same iff *a*_1_≥*a*_2_ and *b*_1_≤*b*_2_.

#### G.4 System implementation

This section describes one implementation of the framework.

The raw text was parsed by Charniak-Johnson parser using David McClosky’s biomedical model [53]. We chose Charniak-Johnson parser because it was convenient in comparing the evaluation with existing systems [54, 55]. But other constituent parsers would also work with little integration effort.

We consider the typing as a critical component of the framework. For example, (1) for relations like phosphorylation, the theme needs to be a noun phrase of type protein or protein part; (2) for triggers like “associate”, the binding relation should not be extracted if its themes’ are not proteins or protein parts; and (3) for triggers like “express” and “detect”, the themes’ type must be gene or mRNA, and the relation is either gene expression or transcription, respectively. This implementation of the framework uses a modified version of BioNex, which was developed based on ideas from [39] and used in RLIMS-P [12]. BioNex can detect semantic types of entities referred by nouns or noun phrases, such as protein, gene, chemical or their part. The type detection is based on considering the head nouns and their suffixes, and comparing them with a predefined list for each type.

Patterns were generated and matched from the parse tree using the tree regular expression [56]. Thus pattern templates were designed using tree regular expression as well. 26 pattern templates were created. To extract the predicate in a consecutive verb group, (e.g., “bind” in “is known to bind”), we looked at the verb phrase subtree and searched for its rightmost children. When the last verb phrase in the group was found, we picked its head.

For the simplification task, we used iSimp, which is a sentence simplifier specifically created for biomedical text [33]. Currently, iSimp can detect six types of simplification constructs: coordination, relative clause, apposition, introductory phrase, subordinate clause, and parenthetical element. It uses shallow parsing and state transition networks to detect all forms of simplifications. The detection of various simplification constructs is based on the chunks (noun phrases, verb groups, and prepositional phrases), and from these, iSimp generates simplified sentences. iSimp also handles nested constructs. For an in-depth description of this process, we refer the reader to [33].

For anaphora resolution, we used JavaRAP, which is based on the algorithm of [57] and implemented by [58]. Other referential relation patterns were defined using tree regular expressions.

The discussion above describes an implementation of the framework. In order to evaluate the framework using the BioNLP-ST GE data, we implemented a relation extraction system for the six events in these data sets. The relation extraction system is obtained from this implementation by specifying the triggers, which were chosen by considering a subset of the trigger words marked in the training set for the six events in the GE 2011 training set. In particular, we chose only frequently occurring verbal trigger words. Note the trigger specifications require only the base form of these verbal triggers (e.g., “phosphorylate” and “interact”). Because this set of triggers are limited in the subcategorization variety, they fall into a handful set of predefined trigger specifications. As a result, we are able to quickly complete the trigger specification for these words.

This relation extraction system implementation is available as a web service accessible: http://research.bioinformatics.udel.edu/ixtractr. Unlike the evaluations conducted in this paper, the web service does not have gene mentions marked in the text as the input. Instead, we integrated an in-house module to detect gene mentions. Because this module only accepts PMIDs as the input rather than full text, the current web service only supports PMID input as well.

## Results and discussion

### A. Results on GE 2011 corpus

After trigger selection, events related to the selected triggers were found to be very frequent in the corpus, covering 81.46% and 78.78% of all events in the training and development sets of the GE 2011 corpus, respectively (Table 2). This intrinsic limitation, however, led to an upper bound of 89.78% and 88.13% in the F-score of our system.

**Table 2:** **Statistics of the data sets after modification**

Events		Training set		Development set
		(%)		(%)
Simple Event	3,165	84.92	923	80.19
Gene_expression	2,094	86.64	614	79.23
Transcription	511	72.59	115	69.28
Protein_catabolism	105	92.11	22	95.65
Phosphorylation	185	94.87	107	95.54
Localization	270	90.91	65	86.67
Binding	874	71.00	380	75.55
*Total*	4,039	81.46	1,303	78.78

Statistics of events with selected triggers on BioNLP-ST 2011 ST GE task. If an event’s argument is within an equivalence relation with *n* members, this event will be counted *n* times. % = Events with selected triggers/All events.

Table 3 summarizes the performance of our system on the training set of the GE 2011 corpus. We provide results for the Simple Event averaging over five events, results for each of the six individual events including Binding, as well as the overall results for all events. Overall, we obtained a global F-score of 77.78% for the Simple Event and 65.14% for the Binding Event. The second part of the results shows the Precision/Recall/F-score when we limited the task to subset events containing only selected triggers. Here, we achieved an F-score of 85.18% for the Simple Event and 79.44% for the Binding Event. We also noted that our system attain a higher precision (> 90%) and a higher recall (> 70%) on this data subset.

**Table 3:** **Evaluation results on the whole, abstract, and full paper collections from the training set of BioNLP-ST 2011 GE task**

	Whole			Abstract			Full		
	P	R	F	P	R	F	P	R	F
**Whole set**									
Simple event	92.40	67.16	77.78	93.11	66.87	77.84	89.36	68.44	77.52
Gene_expression	92.27	69.18	79.07	93.01	68.82	79.11	89.86	70.43	78.96
Transcription	92.43	55.54	69.39	92.88	55.67	69.61	89.66	54.74	67.97
Protein_catabolism	91.01	71.05	79.80	93.10	71.05	80.60	–		
Phosphorylation	97.42	77.44	86.29	97.78	76.74	85.99	95.00	82.61	88.37
Localization	90.43	70.03	78.94	91.24	70.46	79.52	76.92	62.50	68.97
Binding	90.83	50.77	65.14	90.76	50.68	65.04	91.43	51.61	65.98
*Total*	92.08	63.10	74.88	92.60	62.57	74.68	89.61	65.83	75.90
**Subset with selected triggers**									
Simple event	92.29	79.08	85.18	92.98	78.92	85.38	89.36	79.79	84.31
Gene_expression	92.27	79.85	85.61	93.01	79.25	85.58	89.86	81.94	85.71
Transcription	92.00	76.52	83.55	92.37	77.93	84.54	89.66	68.42	77.61
Protein_catabolism	91.01	77.14	83.51	93.10	77.14	84.38	–		
Phosphorylation	97.42	81.62	88.82	97.78	81.48	88.89	95.00	82.61	88.37
Localization	90.04	77.04	83.03	90.83	77.65	83.72	76.92	66.67	71.43
Binding	90.25	70.94	79.44	90.11	70.47	79.09	91.43	75.29	82.58
*Total*	91.88	77.32	83.97	92.35	76.95	83.95	89.61	79.22	84.09

Performance is reported in terms of (P)recision/(R)ecall/(F)-score.

Table 4 summarizes our results on the development set of the GE 2011 corpus. On the whole set, F-scores are reported as 75.10% for the Simple Event and 62.20% for the Binding Event. On the subset, F-scores are 84.27% and 73.55%, respectively. In our experiment, the development set of GE 2011 task contains 1,654 events while the training set contains 4,956 events. Therefore, the numbers on development sets are somewhat lower than those on the training set because a single error or missing case has a higher impact on the overall performance on this smaller data set.

**Table 4:** **Evaluation results on the whole, abstract, and full paper collections from the development set of BioNLP-ST 2011 GE task**

	Whole			Abstract			Full		
	P	R	F	P	R	F	P	R	F
**Whole set**									
Simple event	92.06	63.42	75.10	92.04	65.61	76.61	92.08	61.05	73.42
Gene_expression	92.28	64.77	76.12	91.01	66.75	77.02	93.61	62.88	75.23
Transcription	89.13	49.40	63.57	94.55	57.78	71.72	81.08	39.47	53.10
Protein_catabolism	94.12	69.57	80.00	93.75	71.43	81.08	100.00	50.00	66.67
Phosphorylation	98.77	71.43	82.90	96.77	62.50	75.95	100.00	78.13	87.72
Localization	84.75	66.67	74.63	91.49	70.49	79.63	58.33	50.00	53.85
Binding	91.51	47.12	62.20	86.96	42.94	57.49	98.98	54.80	70.55
*Total*	91.92	58.46	71.47	90.65	57.62	70.46	93.53	59.53	72.76
**Subset with selected triggers**									
Simple event	91.17	78.33	84.27	91.10	78.74	84.47	91.26	77.86	84.03
Gene_expression	91.18	80.78	85.66	89.93	82.51	86.06	92.48	79.10	85.27
Transcription	89.13	71.30	79.23	94.55	73.24	82.54	81.08	68.18	74.07
Protein_catabolism	94.12	72.73	82.05	93.75	71.43	81.08	100.00	100.00	100.00
Phosphorylation	98.77	74.77	85.11	96.77	65.22	77.92	100.00	81.97	90.09
Localization	83.05	75.38	79.03	89.36	79.25	84.00	58.33	58.33	58.33
Binding	90.73	61.84	73.55	85.71	57.02	68.49	98.98	70.29	82.20
*Total*	91.06	73.52	81.36	89.63	71.60	79.61	92.89	76.01	83.61

Performance is reported in terms of (P)recision/(R)ecall/(F)-score.

Table 5 shows the effects of different system components on the overall results of our system. We considered three scenarios: (1) using only the argument and null argument patterns; (2) using also the sentence simplification; and (3) using both sentence simplification and referential relation linking. Note that the result of scenario (3) is the same as in the second part of Table 3. Overall, sentence simplification increased the recall by 23%, while referential relation linking achieved an additional 8% increase. Likewise, results for the development set shows an increase of 22% and an additional 7% in recall by simplification and referential relation, respectively (Table 6). Results in both tables indicated that without increasing the number of patterns, simplification and referential relation linking are helpful in extracting more instances of relations.

**Table 5:** **Comparative results of subset events with selected triggers on the whole, abstract, and full paper collections from the training set of BioNLP-ST 2011 GE task**

	Whole			Abstract			Full		
	P	R	F	P	R	F	P	R	F
**Basic patterns**									
Simple event	93.37	50.74	65.75	93.68	49.30	64.61	92.20	57.17	70.58
Binding	94.87	29.63	45.16	94.54	28.52	43.82	97.14	40.00	56.67
*Total*	93.58	46.17	61.84	93.81	44.44	60.31	92.64	54.97	69.00
**Using simplification**									
Simple event	93.30	73.87	82.45	94.28	73.28	82.46	89.31	76.51	82.42
Binding	92.34	51.03	65.73	92.25	49.81	64.69	92.98	62.35	74.65
*Total*	93.14	68.93	79.23	93.92	67.79	78.75	89.69	74.70	81.51
**Using simplification and referential relations**									
Simple event	92.29	79.08	85.18	92.98	78.92	85.38	89.36	79.79	84.31
Binding	90.25	70.94	79.44	90.11	70.47	79.09	91.43	75.29	82.58
*Total*	91.88	77.32	83.97	92.35	76.95	83.95	89.61	79.22	84.09

Performance is reported in terms of (P)recision/(R)ecall/(F)-score. The third part is reproduced from the second part of Table 3. “Basic patterns” = using pattern templates for argument realization and pattern templates with null argument to generate patterns.

**Table 6:** **Comparative results of subset events with selected triggers on the whole, abstract, and full paper collections from the development set of BioNLP-ST 2011 GE task**

	Whole			Abstract			Full		
	P	R	F	P	R	F	P	R	F
**Basic patterns**									
Simple event	93.01	51.90	66.62	91.67	48.99	63.85	94.42	55.24	69.71
Binding	94.95	24.74	39.25	91.67	22.73	36.42	100.00	28.26	44.07
*Total*	93.32	43.98	59.78	91.67	40.35	56.04	95.17	48.68	64.41
**Using sentence simplification**									
Simple event	92.99	74.76	82.88	92.71	74.70	82.74	93.31	74.83	83.05
Binding	94.59	46.05	61.95	91.67	45.45	60.77	100.00	47.10	64.04
*Total*	93.31	66.39	77.58	92.47	65.08	76.40	94.38	68.08	79.10
**Using sentence simplification and referential relations**									
Simple event	91.17	78.33	84.27	91.10	78.74	84.47	91.26	77.86	84.03
Binding	90.73	61.84	73.55	85.71	57.02	68.49	98.98	70.29	82.20
*Total*	91.06	73.52	81.36	89.63	71.60	79.61	92.89	76.01	83.61

Performance is reported in terms of (P)recision/(R)ecall/(F)-score. The third part is reproduced from the second part of Table 4. “Basic patterns” = using pattern templates for argument realization and pattern templates with null argument to generate patterns.

Table 7 shows results from the test set of 2011 GE tasks. Our system achieves an overall F-score of 72.66% for the Simple Event, as compared to the F-score of 73.90% which was the best score for the Simple Event subtask on the GE 2011 test set [51]. Our system achieves an F-score of 55.57% for the Binding Event, as compared to the F-score of 48.79% which was the best score for the Binding Event subtask on the GE 2011 test set. The best rule-based system achieved F-scores of 70.52% and 36.88% with Simple and Binding events, respectively. Thus, our results compare favorably with those of the top-achieving systems that participated in the GE 2011 task.

**Table 7:** **Results on the (W)hole, (A)bstract, and (F)ull paper collections from the testing set of BioNLP-ST 2011 GE task 1**

Event class	Whole			Abstract			Full		
	P	R	F	P	R	F	P	R	F
Simple event	92.59	59.80	72.66	92.2	56.09	69.75	93.52	71.17	80.83
Gene_expression	91.89	62.18	74.17	91.29	58.03	70.96	93.15	72.86	81.76
Transcription	93.10	46.55	62.07	92.86	47.45	62.80	94.12	43.24	59.26
Protein_catabolism	100.00	66.67	80.00	100.00	64.29	78.26	100.00	100.00	100.00
Phosphorylation	92.52	73.51	81.93	91.51	71.85	80.50	95.12	78.00	85.71
Localization	96.67	45.55	61.92	97.33	41.95	58.63	93.33	82.35	87.50
Binding	89.58	40.28	55.57	89.20	35.40	50.70	90.10	51.12	65.20
*Total*	91.97	54.55	68.48	91.68	50.89	65.45	92.64	64.83	76.28

Performance is reported in terms of (P)recision/(R)ecall/(F)-score.

We would like to note that although Table 3, 4, 5, 6, and 7 show the results on different partitions of the 2011 data sets, the system remains unchanged because the trigger word list (extracted from the training set) remains the same.

### B. Analysis of false positives and negatives on GE 2011 corpus

We randomly chose 50 false positive (FP) cases and 180 false negative (FN) cases with 30 for each event type from the training set of GE 2011 corpora in order to analyze reasons for failure. We identified two major types of errors.

#### B.1 Parsing errors

**Figure 3:**
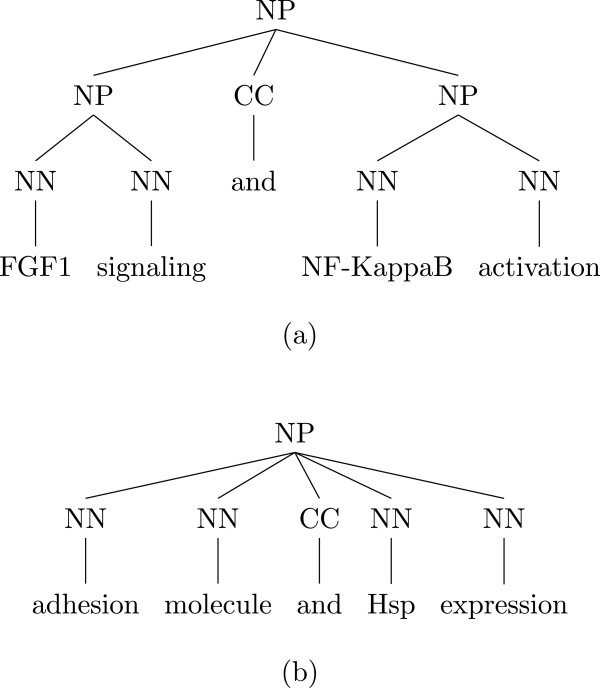
**Two parse trees of coordinations. (a)** Parsing tree of the fragment “FGF1 signaling and NF-KappaB activation”. **(b)** Parsing tree of the fragment “adhesion molecule and Hsp expression”.

A large proportion of failure was due to errors made by the parser. Since the patterns rely on the parser output, the system failed to recognize a true positive in these cases. Some of the parsing errors were due to noun phrase coordinations. Although the parser detected the coordination, the resulting trees could have been shallow or deep. Figure 3 shows two different parse trees of noun phrase coordinations: (a) is correctly parsed, but (b) is not. Flattening the coordination and applying relaxed matching rules could have fixed most of these problems. For coordination simplifications in particular, we could apply noun phrase and verb group similarity rules to detect coordination boundary and transform the subtree from (b) to (a) [33].

**Figure 4:**
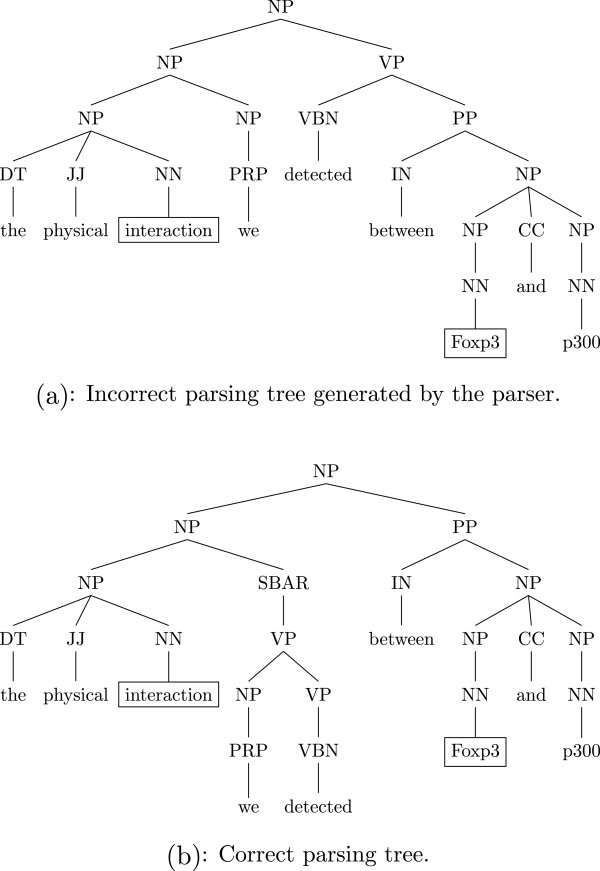
**Two parse trees of the fragment “the physical interaction we detected between Foxp3 and p300”.** Parse tree of the fragment “the physical interaction we detected between Foxp3 and p300”, with target relation <*interaction*_trigger_, Foxp3 _theme_ > highlighted. **(a)** is the incorrect parse tree generated by the parser. **(b)** is the correct parse tree.

Parsing errors also cause simplification errors. Figure 4 shows the parsing subtree of the fragment “the physical interaction we detected between Foxp2 and p300”. If the parse tree were correct, we could remove the relative clause “we detected” in the simplification step and extract the binding relation between “Foxp3” and “p300”, but the incorrect parse tree failed the system. As can be seen, errors in sentence simplification can propagate and cause errors in subsequent processing. Most of the simplification errors are due to incorrect coordination detection. However, overall the number of simplification errors are few, and as can be seen from Table 6, the boost in recall is significantly more than the drop in precision.

#### B.2 Missing pattern templates

Another case of false negatives is due to the trigger word being a noun but not the head of the noun phrase. For example, our pattern templates could be applied for fragments “*transcription of* NP” and “*expression of* NP” but could not be applied for fragments “ of NP_2_” or “ of NP_2_”. We impose such a constraint in order to maintain a high precision. The analysis showed, however, that we could generalize the constraints in the future with some effort, especially in deciding on the words that can head the NPs.

Similarly, we need to generalize null argument structures further. For example, consider the fragment

(33) targets c-Fos *for***degradation**

We have a pattern template using “via” but not “for”. There are a few other cases, where null argument pattern templates could have been applied, but these new templates need to be further checked.

### C. Results and analysis on GE 2013 corpus

Table 8 shows the results for the same six events of the GE 2013 test set. We still used the trigger list from the 2011 training set. Thus, the system was the same one used on the 2011 task, without any changes made for the evaluation on the new corpus.

**Table 8:** **Evaluation results from the training, development, and testing sets of BioNLP-ST 2013 GE task 1**

Event class	P	R	F
**Training set**			
Simple event	86.75	72.20	78.81
Binding	88.95	64.83	75.00
*Total*	87.12	70.82	78.13
**Development set**			
Simple event	89.52	71.73	79.64
Binding	93.58	64.42	76.31
*Total*	90.68	69.39	78.62
**Testing set**			
Simple event	90.48	71.80	80.07
Binding	71.80	52.39	60.58
*Total*	85.27	66.05	74.44

Performance is reported in terms of (P)recision/(R)ecall/(F)-score.

The system achieves F-scores of 80.07% for the Simple Event and 60.58% for the Binding Event on the GE 2013 test set with an overall F-scores of 74.44% on the 2013 GE task. These scores compare favorably with the top-ranking systems in the 2013 GE task^a^[59]. Our system achieves the highest scores for Simple Event and Overall. However there are two participated systems (BioSEM and HDS4NLP), which have better scores for Binding Event. In comparison with these systems, our system’s strength lies in its precision, achieving 85.52%, whereas, the precision of these systems ranges from 72.90% to 80.99%.

**Figure 5:**
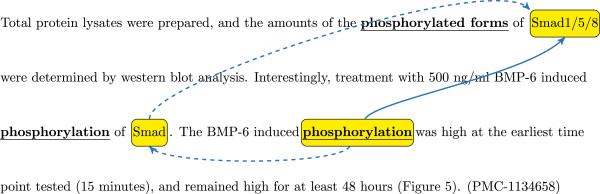
**Sample use of ellipses in the paragraph.** For the trigger “phosphorylation” in the third sentence, the author neglected to mention the theme because it can be inferred from the context: (1) the previous sentence also mentions this “BMP-6 induced phosphorylation”, but its theme has a general term “Smad”, and (2) the actual proteins “Smad1/5/8” are clearly specified in the first sentence.

The testing set of the GE 2013 task is not available to the public, hence we cannot directly examine the results. Instead, we conducted experiments on the training and development sets. Although the results on these two sets are consistent with the corresponding results on 2011 corpora, we noticed some differences between the GE 2013 and 2011 corpora: the former is completely comprised of full-length articles, whereas the latter is mostly made up of abstracts. We also observed that in the full-length articles, certain information is repeatedly mentioned within a single section, therefore there is significant use of ellipses in such sections. For instance, consider the example from the GE 2013 development corpus in Figure 5.

For the trigger “phosphorylation” in the third sentence, the author neglected to mention the theme because it can be inferred from the context: (1) the previous sentence also mentions this “BMP-6 induced phosphorylation”, but its theme has a general term “Smad”, and (2) the actual proteins “Smad1/5/8” are clearly specified in the first sentence. As a result, to infer the theme of the trigger “phosphorylation” in the third sentence, we not only need the syntax information, but also the discourse-level processing.

Note that the system used in this evaluation remains the same as the one that was used on the GE 2011 task. No changes were made to accommodate any differences between the GE 2011 and 2013 corpora. The focus in this framework is on the patterns and hence almost all processing is syntax-based. While some of our earlier work on relation extraction has integrated discourse-level processing with syntax-based patterns [60], the integration of such discourse-level processing is beyond the scope of this work. However, examples as above suggest that the need for discourse-level processing may be important for full-length based extraction. We intend to investigate incorporating the generalized discourse-level processing into our framework in the future, so that it can be useful for full-text based extraction.

## Conclusions

In this work, we have designed a framework for development of biomedical relation extraction systems. The framework requires as input only a list of triggers and their specifications to retrieve relations of interest. It utilizes linguistic generalizations that help speed up the development process by proposing various lexico-syntactic patterns as well as improve the performance, particularly the recall, by making use of sentence simplification and referential relations.

To evaluate the framework, we developed a relation extraction system, which was produced using general resources and the only aspect specific to the evaluation was the selection of trigger words that appear in the corpus. Except for the specification of triggers, other aspects (parser, typing system, simplification, pattern matching system) are general purpose systems that already existed. The fact that only the specification of the triggers is required from domain experts, together with the fact that no training set is required, meets our goals for developing the framework: ability to create effective relation extraction systems for new relations where resources (e.g., annotated corpus or database) are not publicly available.

We evaluate the performance of the system by producing a relation extraction system and evaluating it on the BioNLP-ST 2011 and 2013 GE tasks. The system achieved F-scores of 68.48% on the GE 2011 test set, and 74.44% on the GE 2013 test set. Our analysis shows that we can achieve high precision and good recall with the range of patterns automatically generated from triggers and that simplification and referential relation linking serve to increase the recall while maintaining the precision.

In the future, we would like to extend the framework in two ways. So far, we only considered the triggers that are verbs and their derived forms. Next, we would like to account for triggers that are primarily nouns or adjectives. Also, we would like to extend the framework to take complex entities (e.g., relations themselves) as arguments rather than just simple entities (e.g., genes or proteins).

We are developing systems for additional relations. In general, it is a challenging task to identify all the triggers for the relation and to complete their specifications. This study demonstrates a generalizable relation extraction framework that can be quickly implemented for new relations, initially focusing on a few triggers that appear frequently. While not accounting for a long tail of less frequent triggers, our framework allows additional trigger specifications to be added with little impact on the existing trigger list. Thus as new triggers are found, they can be integrated in the system. Using the framework and this approach, we have developed a system for miRNA-target extraction. Preliminary evaluation based on an in-house corpus of 200 abstracts shows an F-score of the system over 90% (manuscript in preparation). We would like to use the experience in developing this and other relation extraction to design a process involving user interaction in generating trigger specifications for new relations. In general, the specification of a trigger needs both domain knowledge as well as linguistic knowledge. The domain expert will be able to suggest the trigger words for a relation, whereas linguistic knowledge will be more useful in preparing the trigger specifications of sub-categorization, thematic roles, etc.

In our framework, we already have a predefined set of subcategorization frames and thematic roles that can be utilized in the specifications. This can be used to engage the user in the interactive process. At the beginning, the users who are domain experts will provide a list of trigger words. Then the process will derive various forms of triggers using the linguistic knowledge and ask users to choose. If necessary, the process will use these triggers to generate simple examples for the users to confirm which predefined specification should be associated to the trigger. The whole process will communicate with users in an interactive way, which we expect is able to further speed up the development of new relation extraction systems.

## Endnote

^a^ Simple Event includes Phosphorylation as well, same as in the BioNLP-ST 2011 GE Task 1.
